# 1592. Real-World Utilization and Effectiveness of Long-Acting Cabotegravir + Rilpivirine Among People with HIV with Detectable Viral Loads at Initiation: Trio Cohort Study

**DOI:** 10.1093/ofid/ofad500.1427

**Published:** 2023-11-27

**Authors:** Rick A Elion, Andrew Frick, Janna Radtchenko, Gayathri Sridhar, Supriya Sarkar, Joseph J Eron, Karam Mounzer, Jean A van Wyk, Vani Vannappagari

**Affiliations:** Trio Health, Louisville, Colorado; Trio Health, Louisville, Colorado; Trio Health, Louisville, Colorado; ViiV Healthcare, Fairfax, Virginia; ViiV Healthcare, Fairfax, Virginia; University of North Carolina at Chapel Hill School of Medicine, Chapel Hill, North Carolina; Philadelphia Fight Community Health Centers, Philadelphia, Pennsylvania; ViiV Healthcare, Brentford, UK, Brentford, England, United Kingdom; ViiV Healthcare, Fairfax, Virginia

## Abstract

**Background:**

Cabotegravir+Rilpivirine (CAB+RPV) is the first FDA-approved complete long-acting (LA) injectable antiretroviral therapy (ART) for treatment of HIV-1 infection among ART-experienced, virologically suppressed (VL < 50 c/mL) people with HIV (PWH). We assessed utilization and effectiveness among PWH with VL ≥ 50 c/mL at initiation in real world clinical setting.

**Methods:**

Adult PWH who received ≥ 1 CAB+RPV injections through March 2023 with VL ≥ 50 c/mL at initiation were identified using electronic medical records from Trio Health HIV Network. Results were stratified by VL ≥ 50 c/mL and ≥ 200 c/mL at initiation. Genotypic resistance data prior to initiation of CAB+RPV LA was available for a subset of individuals, with resistance interpretation described using Stanford HIVdb algorithm.

**Figure:**
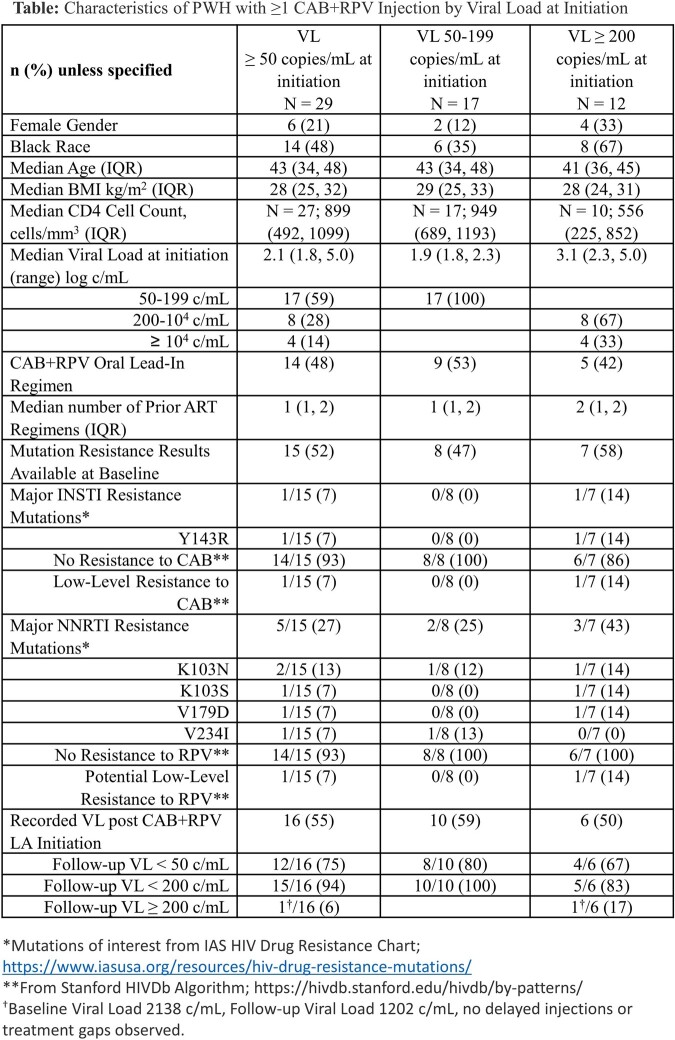

**Results:**

Among 329 PWH with ≥ 1 CAB+RPV injections, 29 (9%) had VL ≥ 50 c/mL at initiation. All were treatment-experienced and 12 (41%) had VL ≥ 200 c/mL, median log VL 3.1 (IQR: 2.3, 5.0) at initiation; 21% were women, 48% were Black, median age was 43 (IQR: 34, 48), and 41% had a BMI ≥ 30.

At the time of analysis, all individuals remained on CAB+RPV LA with median follow-up of 4.0 months (IQR: 2.2, 9.2) with median 3 injections (IQR 1, 5); 20 individuals had ≥ 2 injections and 16 had ≥ 3, with 80/86 (93%) follow-up injections administered within target window. Median time from first to second injection was 32 days (IQR: 30, 36), and median time from second to third injection was 59 days (IQR: 30, 62). Among the 16 individuals with ≥ 3 injections, 12 were on every-2-month dosing schedule.

Sixteen (55%) individuals had a follow-up VL. Of them, the last VL was < 50 c/mL in 12 individuals (75%), < 200 c/mL in 15 (94%), and 1 with VL 1202 c/mL (pre-index VL 2138 c/mL).

Historical HIV genotype results were available for 15 individuals (52%). One individual had low-level resistance to CAB based on Y143R mutation and this individual had a follow-up VL < 50 c/mL. Five individuals had NNRTI mutations, one with low-level resistance to RPV and a follow-up VL < 200 c/mL.

**Conclusion:**

This real-world data of individuals who received ≥ 1 CAB+RPV LA injections with VL ≥ 50 c/mL at initiation demonstrated high rates of virologic suppression. Future analysis with longer follow-up will allow for evaluation of long-term outcomes.

**Disclosures:**

**Rick A. Elion, MD**, Gilead Sciences: Advisor/Consultant|Gilead Sciences: Grant/Research Support|Proteus: Grant/Research Support|ViiV Healthcare: Advisor/Consultant|ViiV Healthcare: Grant/Research Support **Andrew Frick, MS**, Trio Health Inc.: Employee **Janna Radtchenko, MBA**, Trio Health: Employee **Gayathri Sridhar, MBBS, MPH, PhD**, GlaxoSmithKline: Stocks/Bonds|ViiV Healthcare: Full Time Employee **Supriya Sarkar, PhD, MPH**, ViiV Healthcare: Employee **Joseph J. Eron, MD**, Gilead Sciences: Advisor/Consultant|Gilead Sciences: Grant/Research Support|Janssen: Advisor/Consultant|Janssen: Grant/Research Support|Merck: Advisor/Consultant|ViiV Healthcare: Advisor/Consultant|ViiV Healthcare: Grant/Research Support **Karam Mounzer, MD**, Clinical Care Options: Speakers Bureau|Epividian: Advisor/Consultant|Gilead Sciences: Advisor/Consultant|Gilead Sciences: Grant/Research Support|Gilead Sciences: Speakers Bureau|Janssen: Advisor/Consultant|Janssen: Grant/Research Support|Janssen: Speakers Bureau|Merck: Advisor/Consultant|Merck: Grant/Research Support|Merck: Speakers Bureau|Prime: Speakers Bureau|Simply Speaking: Speakers Bureau|ViiV Healthcare: Advisor/Consultant|ViiV Healthcare: Grant/Research Support|ViiV Healthcare: Speakers Bureau **Jean A. van Wyk, MBChB, MFPM**, ViiV Healthcare Ltd: Stocks/Bonds **Vani Vannappagari, MBBS, MPH, PhD**, GlaxoSmithKline: Stocks/Bonds|ViiV Healthcare: Employee

